# Longitudinal dietary trajectories from pregnancy to 3 years post delivery in women with obesity: relationships with adiposity

**DOI:** 10.1002/oby.23706

**Published:** 2023-03-06

**Authors:** Kathryn V. Dalrymple, Christina Vogel, Angela C. Flynn, Paul T. Seed, Keith M. Godfrey, Lucilla Poston, Hazel M. Inskip, Sarah R. Crozier

**Affiliations:** ^1^ Department of Women and Children's Health School of Life Course and Population Sciences, King's College London London UK; ^2^ MRC Lifecourse Epidemiology Centre University of Southampton Southampton UK; ^3^ NIHR Southampton Biomedical Research Centre University of Southampton and University Hospital Southampton NHS Foundation Trust Southampton UK; ^4^ NIHR Applied Research Collaboration Wessex Southampton Science Park Southampton UK; ^5^ Department of Nutritional Sciences School of Life Course and Population Sciences, King's College London London UK

## Abstract

**Objective:**

The study aim was to examine the relationships between longitudinal dietary trajectories from early pregnancy to 3 years post delivery and adiposity measures in women with obesity.

**Methods:**

The diets of 1208 women with obesity in the UPBEAT (UK Pregnancy Better Eating and Activity Trial) study were assessed using a food frequency questionnaire (FFQ) at 15^+0^ to 18^+6^ weeks’ gestation (baseline), 27^+0^ to 28^+6^ weeks’ gestation, and 34^+0^ to 36^+0^ weeks' gestation, as well as 6 months and 3 years post delivery. Using factor analysis of the baseline FFQ data, four dietary patterns were identified: fruit & vegetable, African/Caribbean, processed, and snacking. The baseline scoring system was applied to the FFQ data at the four subsequent time points. Group‐based trajectory modeling was used to extract longitudinal dietary pattern trajectories. Using adjusted regression, associations between dietary trajectories and log‐transformed/standardized adiposity measures (BMI and waist and mid‐upper arm circumferences) at 3 years post delivery were examined.

**Results:**

Two trajectories were found to best describe the data for the four individual dietary patterns; these were characterized as high and low adherence. A high adherence to the processed pattern was associated with a higher BMI (β = 0.38 [95% CI: 0.06–0.69]) and higher waist (β = 0.35 [0.03–0.67]) and mid‐upper arm circumferences (β = 0.36 [0.04–0.67]) at 3 years post delivery.

**Conclusions:**

In women with obesity, a processed dietary pattern across pregnancy and 3 years post delivery is associated with higher adiposity.

**Study Importance**

**What is already known?**

- Rates of maternal obesity are increasing worldwide; this has significant health implications for both the mother and her child.
- So that effective public health interventions can be implemented, it is important to identify and understand the relationship between modifiable determinants and the development of obesity.
- There is a paucity of research exploring the relationship between diet during and post pregnancy and longer‐term obesity outcomes in women.

**What does this study add?**

- We have modeled longitudinal dietary pattern trajectories across the antenatal and postnatal period in a cohort of women with obesity. We found that trajectories of dietary intake tracked from early pregnancy to 3 years post delivery.
- Women who continually followed a diet high in processed foods had higher adiposity outcomes at 3 years post delivery. These women were also more likely to have lower educational attainment, to have higher neighborhood deprivation, and to smoke during pregnancy.

**How might these results change the direction of research or the focus of clinical practice?**

- These findings support the development of policies that aim to improve unhealthy food environments, particularly in more deprived or socioeconomically disadvantaged areas, as well as public health strategies focused on improving nutrition behaviors before and between pregnancies.

## INTRODUCTION

The global obesity epidemic is a major public health challenge across all stages of the life course [1]. Excess weight gain is associated with comorbidities including hypertension, insulin resistance, and physical problems, which impact quality of life and health outcomes. In the UK, obesity is the most common medical condition among pregnant women [2]. Nearly a quarter of women start pregnancy with body mass index (BMI) ≥30 kg/m^2^, and prevalence is higher in those living in deprived areas [3]. A concerning implication of obesity in pregnancy lies in the health consequences for both the mother and the child. Acutely, the mother is at risk of gestational diabetes mellitus (GDM), excessive gestational weight, and adverse outcomes in labor, associated with fetal macrosomia [4]. In the longer term, maternal obesity is associated with the development of cardiovascular disease and type 2 diabetes [5], and the children are themselves at greater risk of developing obesity [6].

Obesity is a complex health issue with no single determinant. In England, the prevalence is higher in more socioeconomically disadvantaged communities and among population groups of Black and Asian ethnicities [7], as well as among those who are physically inactive [8]. Unhealthy environmental exposures, which are more common in disadvantaged areas [9], also are associated with obesity development. These include limited infrastructure to support active travel, such as foot or cycle paths, and abundant access to takeout and processed foods. Consumption of energy‐dense and ultraprocessed foods is one of the most prominent factors associated with the development of obesity [10]. There is also strong evidence describing the relationships of maternal diet and lifestyle in pregnancy [4] and postnatally [11] with offspring obesity. Less well understood is the evidence demonstrating that diet during and after pregnancy is a contributor to increased weight gain during the reproductive years, acting in part through excessive gestational weight gain and subsequent postpartum weight retention [12]. Several studies have modeled longitudinal dietary trajectories across the antenatal and postnatal period [13, 14]. These investigations have assessed the stability of diet over time by converting the continuous dietary indicator into an ordered categorical variable at each assessment point [14] or applied latent class transition analysis [13]. However, none has explored relationships between longitudinal dietary intake and adiposity in the mother. A strength of latent class analysis is that it classifies individuals within a given data set into subgroups that follow similar longitudinal trajectories over time [15]. Such modeling of dietary data can then be used to identify relationships between trajectories of diet and sociodemographic characteristics and adiposity measures. Of practical use in health care settings, this approach can identify patterns of diet that persist over time and that are suitable targets for intervention.

To support obesity prevention, it is important to identify population groups affected by multiple exposures and focus research efforts on those most at risk of obesity‐related poor‐health outcomes. Therefore, we have modeled dietary pattern trajectories from early pregnancy to 3 years post delivery using data from the UK Pregnancy Better Eating and Activity Trial (UPBEAT), an ethnically diverse cohort of women with prepregnancy obesity predominantly living in socially deprived areas [16]. The aim of this analysis was to describe longitudinal dietary pattern trajectories using group‐based trajectory modeling (GBTM) and to (1) explore associations between trajectories and sociodemographic characteristics and (2) describe the associations between trajectories and maternal adiposity measures 3 years after giving birth among a predominantly socioeconomically disadvantaged cohort of women.

## METHODS

### Study setting

This study is a longitudinal secondary analysis from the UPBEAT study, a multicenter randomized controlled trial that aimed to investigate the effect of an 8‐week lifestyle (diet and physical activity) intervention in 1555 pregnant women with obesity [16]. Participants were from multiethnic, socioeconomically disadvantaged backgrounds and they were recruited between March 2009 and June 2014 from eight UK inner‐city settings. Full details of the study, including inclusion and exclusion criteria, have been published [16]. Women were randomized to either standard antenatal care or the intervention arm between 15^+0^ and 18^+6^ weeks’ gestation (baseline), and all participants provided written informed consent. Women were followed up between 27^+0^ and 28^+6^ weeks’ gestation (end point of the intervention), between 34^+0^ and 36^+0^ weeks’ gestation (late pregnancy), and 6 months and 3 years post delivery. At delivery, neonatal characteristics and birth outcomes were recorded. The intervention had no effect on the primary outcomes: prevalence of GDM or large for gestational age infants. However, there were several effects on secondary outcomes at the end of the 8‐week intervention, including reductions in gestational weight gain, in sum of skinfold thicknesses, in dietary glycemic load, and in saturated fat intake and an increase in self‐reported physical activity [16].

### Maternal data

At baseline, details on maternal age, ethnicity, socioeconomic status (SES; assessed by Index of Multiple Deprivation [IMD]) [17], smoking status (any vs. none in pregnancy), parity, and measures of body composition were recorded. Weight was measured without shoes and heavy clothing to the nearest 0.1 kg using Class III scales. Height was measured to the nearest centimeter using routinely collected data, and BMI was then calculated. Circumferences of the hip, mid‐upper arm (MUA), thigh, and waist were measured in triplicate, and the mean was calculated. Weight and circumference measurements were repeated at the end point of the intervention, in late pregnancy, and at 6 months and 3 years post delivery.

### Food frequency questionnaire

At each visit, diet was assessed in all participants using a 50‐item food frequency questionnaire (FFQ) adapted from the UK arm of the European Prospective Investigation into Cancer Study [18]. Questionnaires were administered by research midwives. Response options for each food item included the following: less than once per month, 1 to 3 times per month, once a week, 2 to 4 times per week, 5 to 6 times per week, once a day, 2 to 3 times per day, 4 to 5 times per day, and 6+ times per day. Using the average daily frequency of the 50 food items in the questionnaire, factor analysis with orthogonal varimax rotation was performed on the baseline data. After completion of principal component analysis to identify dietary patterns that capture independent variability in diet, the number of components retained was chosen using scree plots. Orthogonal rotation was used to improve interpretation. Dietary pattern labels were chosen based on foods with the highest factor loadings (≥0.25). Full details of this analysis have been published [19]; four distinct baseline dietary patterns were defined, namely “Fruit & vegetable,” “African/Caribbean,” “Processed,” and “Snacking.” The scoring system derived at baseline was applied to the FFQ data at the subsequent four assessment time points [20].

### Trajectory modeling

Time point assessments were converted to mean gestational age from baseline (e.g., baseline [15–18 weeks' gestation] was coded as 0 weeks, and late pregnancy [34–36 weeks' gestation] was coded as 20 weeks; Supporting Information Table S1). GBTM was used to identify latent classes of the dietary trajectories (traj command in Stata version 15.0, StataCorp LLC). GBTM is a semiparametric mixture model, with the error variance assumed to be the same for all classes and all time points [21]. This method is able to handle missing data under the missing at random assumption by fitting models using maximum likelihood estimation [22]. As the dietary variables were continuous and normally distributed, a censored normal model was used. For each model applied to the data, it cannot be assumed that all classes follow a similar longitudinal change over time. Therefore, when fitting GBTM it is advised to model trajectories as either intercept, linear, quadratic, or cubic functions. Nonsignificant cubic and quadratic terms are removed from the model, although linear parameters for a trajectory/class can be retained irrespective of significance as long as the model Bayesian information criterion (BIC; a measure of model fit) is lower than when an intercept parameter is used [23]. This process is then repeated until there is no evidence of an improvement in model fit assessed by BIC. This study adheres to the Guidelines for Reporting on Latent Trajectory Studies checklist [24] (Supporting Information Table S1); following the guidelines in the checklist, as the number of latent classes is unknown, we used a forward modeling approach from one to three classes. After fitting the one‐class model, we incrementally added an extra class and investigated the model fit criteria discussed subsequently. To identify the appropriate number of classes, each model was assessed using likelihood‐based statistics and statistics on classifying individuals.

### Likelihood‐based and classification statistics

As mentioned earlier, BIC is a test statistic for model selection, and a value closer to zero indicates a better model fit. Relative entropy should be close to one and is a measure of model classification uncertainty [15]. The ratio of the odds of correct classification (OCC) and the average posterior probability assignment (APPA) are class specific. The OCC should ideally be >5 [15], and it is the ratio of the odds of correctly classifying into each group on the basis of estimated class membership proportions and the maximum probability classification rule. The APPA is calculated as the average posterior probability of belonging to a class over all the individuals assigned to that class; the APPA should be >70% [15]. The minimum percentage of participants assigned to each class should be 5% [22]. The optimal number of classes selected was based on the lowest BIC and satisfactory values for the remaining criteria. Using the same criteria, we also confirmed the latent class trajectories using growth mixture modeling (GMM; gllamm command, Stata 15.0). Unlike GBTM, GMM is a form of latent class growth analysis that allows for random effects [25]. GMM estimates a mean growth curve for each class and uses random effects to summarize individual differences within a class.

### Statistical analysis

For descriptive statistics, binary and categorical variables are presented using counts and percentages. The distributions of continuous variables were assessed using coefficients of skewness and then summarized by means and standard deviations (SD) for normally distributed variables or medians and interquartile ranges (IQR) for non‐normally distributed variables. As there was no effect of the UPBEAT intervention on the dietary trajectories or on the outcomes of interest (Supporting Information Table S2) [26], the data have been analyzed as one single cohort, with adjustment for randomization arm. Once the optimal number of trajectories that best described the data was identified, adjusted regression, using direct acyclic graph‐derived confounders (IMD score, parity, ethnicity, physical activity, maternal age, and intervention arm; Supporting Information Figure S1), was used to examine the relationships between the dietary trajectories and adiposity outcomes. Outcomes were all continuous (BMI and waist, hip, thigh, and MUA circumferences) and were log transformed before analyses. For ease of interpretation, these values were standardized to a mean of 0 and an SD of 1 to allow comparison between measures.

## RESULTS

**FIGURE 1:**
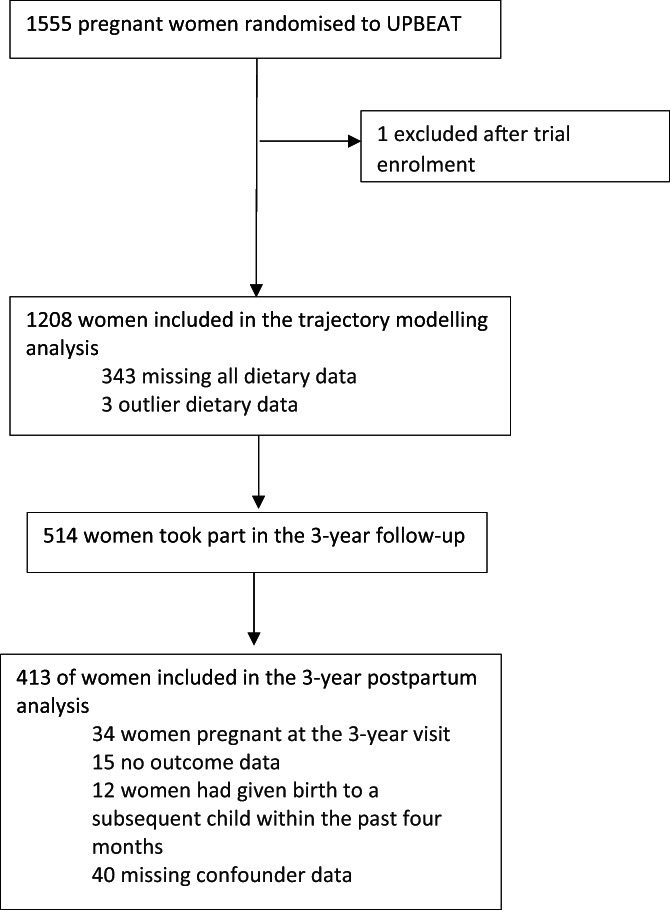
Study flow diagram. UPBEAT, UK Pregnancy Better Eating and Activity Trial

Of 1555 women randomized to UPBEAT, 1211 provided dietary data at a minimum of one time point. Three women were removed from the dietary analysis because of outlying dietary pattern data (± ≥4 SD from the mean); 1208 were included in the longitudinal trajectory modeling. A total of 514 women took part in the 3‐year post‐delivery visit, of whom 413 were included in the final analysis (*n* = 55 had missing outcome/confounder data, *n* = 34 were excluded because they were pregnant at the 3‐year visit, and *n* = 12 women had given birth to a child within the last 4 months; study flowchart in Figure 1). For the 1208 included in the longitudinal dietary analysis, the average age at recruitment was 30.6 (SD 5.4) years, and the median BMI was 34.9 kg/m^2^ (IQR 32.7–38.4). Almost two thirds (64%) of the cohort described themselves as White, 24% as Black, 7% as Asian, and 5% as another ethnicity. The majority (77%) lived in disadvantaged neighborhoods with IMD quintile scores of 4 or 5 (most deprived). Almost half (44%) were nulliparous, and 16% smoked during their pregnancy (including those who gave up in the first trimester). A quarter (24%) of women developed GDM in their pregnancy (Table 1). In comparison with those not included, women included in the final analysis (*n* = 413) were more likely to have a lower BMI at baseline and to have spent longer in full‐time education, and they were less likely to smoke in pregnancy, to be older, and to be nulliparous (Supporting Information Table S3). Baseline dietary pattern scores were similar for those included and not included in this analysis (Supporting Information Table S3).

**TABLE 1:** Baseline demographic characteristics for the UPBEAT cohort and by dietary trajectory using group‐based trajectory modeling

	Total cohort	Fruit & vegetable		African/Caribbean		Processed		Snacking	
	(*N* = 1208)	Low (*n* = 1001)	High (*n* = 207)	Low (*n* = 1066)	High (*n* = 142)	Low (*n* = 1051)	High (*n* = 157)	Low (*n* = 1019)	High (*n* = 189)
Age (y)	30.6 (5.4)	30.3 (5.5)	31.8 (4.9)	30.5 (5.5)	31.4 (5.2)	30.9 (5.3)	28.4 (5.6)	30.6 (5.4)	30.4 (5.4)
BMI (kg/m^2^)	34.9 (32.7‐38.4)	34.9 (32.7‐38.4)	35.2 (32.8‐38.1)	34.9 (32.7‐38.2)	35.0 (33.1‐39.0)	34.9 (32.7‐38.0)	36.1 (33.2‐41.0)	35.0 (32.7‐38.5)	34.5 (32.5‐37.3)
Ethnicity									
White	775 (64%)	652 (65%)	123 (59%)	762 (72%)	13 (9%)	647 (62%)	128 (82%)	632 (62%)	143 (76%)
Black	286 (24%)	232 (23%)	54 (26%)	174 (16%)	112 (79%)	275 (26%)	11 (7%)	261 (26%)	25 (13%)
Asian	83 (7%)	65 (7%)	18 (9%)	73 (7%)	10 (7%)	75 (7%)	10 (6%)	70 (7%)	13 (7%)
Other	64 (5%)	52 (5%)	12 (6%)	57 (5%)	7 (5%)	54 (5%)	8 (5%)	56 (5%)	8 (4%)
IMD score^a^									
Least deprived	51 (4%)	42 (4%)	9 (4%)	51 (5%)	—	46 (4%)	5 (3%)	43 (4%)	8 (4%)
2	88 (7%)	72 (7%)	16 (8%)	85 (8%)	3 (2%)	74 (7%)	14 (9%)	70 (7%)	18 (10%)
3	139 (12%)	119 (12%)	20 (10%)	129 (12%)	10 (7%)	128 (12%)	11 (7%)	120 (12%)	19 (10%)
4	412 (34%)	332 (33%)	80 (39%)	365 (34%)	47 (33%)	368 (35%)	44 (28%)	356 (35%)	56 (30%)
Most deprived	515 (43%)	433 (44%)	82 (39%)	433 (41%)	82 (58%)	432 (41%)	83 (53%)	427 (42%)	88 (46%)
Years in full‐time education	14.8 (2.9)	14.7 (2.8)	15.4 (3.1)	14.8 (2.8)	14.7 (3.4)	15.0 (2.9)	13.9 (2.6)	14.8 (2.9)	14.8 (0.28)
Nulliparous	527 (44%)	442 (44%)	85 (41%)	493 (46%)	34 (24%)	456 (43%)	71 (45%)	449 (44%)	78 (41%)
Smoking in pregnancy	191 (16%)	167 (17%)	24 (12%)	181 (17%)	10 (7%)	155 (15%)	36 (23%)	156 (15%)	25 (13%)
GDM^b^	252 (24%)	214 (25%)	38 (21%)	213 (23%)	39 (33%)	218 (24%)	24 (26%)	219 (25%)	33 (19%)
LGA^c^	98 (8%)	78 (8%)	20 (10%)	85 (8%)	13 (9%)	88 (9%)	10 (7%)	84 (8%)	14 (7%)

*Note*: Data are presented as mean (SD), median (IQR), or *N* (%).

Abbreviations: GDM, gestational diabetes mellitus; IMD, indices of multiple deprivation; LGA, large for gestational age; UBEAT, UK Pregnancy Better Eating and Activity Trial.

^a^ IMD quintiles are calculated for the region of residence, by fifths of the population. UK‐wide scores were developed by reconciling Scottish data to English norms.

^b^ GDM diagnosis by International Association of Diabetes and Pregnancy Study Groups criteria at 27^+0^ to 28^+6^ weeks' gestation.

^c^ Customized birth weight centile adjusting for maternal height and weight, ethnicity, parity, and sex of the infant.

Four distinct dietary patterns were identified by factor analysis at baseline, “fruit & vegetable,” “African/Caribbean,” “processed,” and “snacking.” The full list of factor loadings for each pattern has been published previously [19]. Briefly, the fruit & vegetable pattern comprised a high intake of fruit (fresh, citrus, tropical, and dried), vegetables (green, root, and salad), and yogurt. The African/Caribbean pattern contained high intakes of meat (red and white), cassava, fish, rice (including pilau, fried, or jollof rice), and plantain. The “processed” pattern had high intakes of chocolate, crisps (potato chips), green vegetables, potatoes (including chips/fries), processed meat and meat products, root vegetables, squash, and fizzy (carbonated) drinks (including sugar‐free alternatives). The “snacking” pattern comprised high intakes of biscuits, cookies, cakes, pastries, chocolate, full‐fat cheese, and sweets.

Each participant was assigned a score for each dietary pattern at the five time points. Participants with a higher score for a pattern followed that pattern more closely, whereas those with a low score did not. The class 1 trajectories for each of the dietary patterns are shown in Supporting Information Figure S2. These trajectories show that adherence to the specific pattern decreased during pregnancy and increased again by 6 months/3 years post delivery (Supporting Information Table S4). GBTM was performed on each of the four dietary patterns, and a two‐trajectory group was found to best describe the data for the four individual dietary patterns (Supporting Information Tables S5–S8). These findings were confirmed using GMM (Supporting Information Tables S9–S12). There was also a strong agreement between the two methods and individual class assignment (Spearman correlations = 0.97–0.99; Supporting Information Table S13). There was also crossover between the trajectory groups (Supporting Information Table S14); for example, 16% of those in the high fruit & vegetable group were also in the high African/Caribbean group, and 23% of those in the high snacking group were also in the high fruit & vegetable group.

**FIGURE 2:**
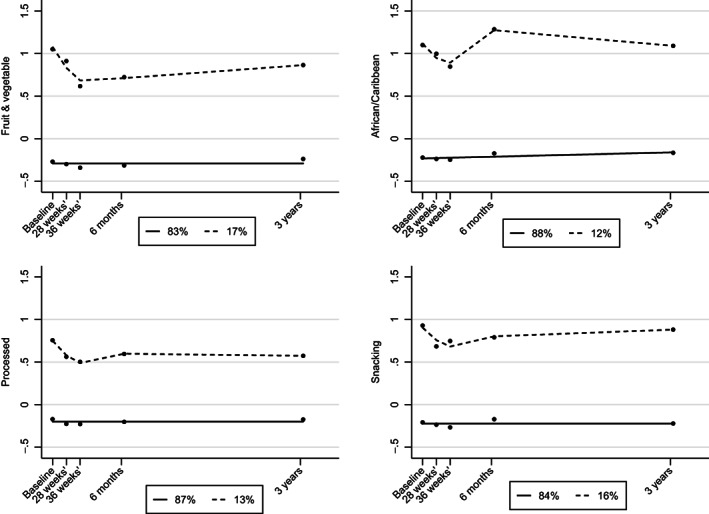
Dietary pattern trajectories, using group‐based trajectory modeling, from early pregnancy to 3 years post delivery. Each graph illustrates the dietary pattern score on the y‐axis with high (dash line) and low adherence (solid line) for the individual dietary patterns.

For the African/Caribbean pattern, although the three‐class model had a lower BIC and satisfactory group membership, the entropy and OCC were lower than the two‐class model. Also, outcome data were available for only 20 women in the third group, which was considered too few for the final part of the analysis. A total of 17%, 12%, 13%, and 16% of women had high adherence longitudinally to the four patterns, respectively (Table 1 and Figure 2). For the high trajectory groups for each of the dietary patterns, adherence decreased during pregnancy and remained lower than the baseline scores up to 3 years post delivery, except for the African/Caribbean pattern (Figure 2). The individual trajectories are shown in Supporting Information Figure S3.

Comparisons were made between women with high adherence to each of the dietary pattern trajectories and those with low adherence. For the fruit & vegetable pattern, women with a higher longitudinal adherence score were more likely to be older and to have spent longer in full‐time education, and they were less likely to have smoked in pregnancy (Table 1). For the African/Caribbean pattern, women with high adherence were more likely to be older, to be of Black ethnicity, to have a higher IMD (more deprived), and to have been diagnosed with GDM, and they were less likely to be nulliparous and to have smoked in pregnancy. For the processed pattern, women with high adherence were more likely to be younger, to have a higher early‐pregnancy BMI, to be of White ethnicity, to have a higher IMD, to have spent less time in high education, and to have smoked in pregnancy. For the snacking pattern, women with high adherence were more likely to be of White ethnicity.

Table 2 shows a summary of adiposity outcomes by trajectory group at 3 years post delivery. For the fruit & vegetable pattern, those with high scores had a lower BMI. For the African/Caribbean pattern, those with high scores had a higher BMI and MUA, hip, and thigh circumferences. For the processed pattern, women with a high score had a higher BMI and waist, MUA, and hip circumferences. For the snacking pattern, women with a high score had a lower BMI and waist and MUA circumferences and a higher hip circumference. Table 3 shows the adjusted regression coefficients for adiposity outcomes for high versus low adherence to a dietary trajectory. Women with high longitudinal adherence to the processed pattern from early pregnancy to 3 years post delivery were more likely to have a higher BMI (β = 0.38 SD [95% CI: 0.06–0.69]) and higher waist (β = 0.35 SD [0.03–0.67]) and MUA (β = 0.36 SD [0.04–0.67]) circumferences at 3 years post delivery. No differences were observed for the other three patterns.

**TABLE 2:** Adiposity outcomes at 3 years post partum from UPBEAT women by dietary pattern trajectory obtained using group‐based trajectory modeling

		BMI (kg/m^2^)	Waist circumference (cm)	MUA circumference (cm)	Hip circumference (cm)	Thigh circumference (cm)
Total cohort	(*N* = 453)	35.2 (32.4‐39.9)	103 (96.0‐112.1)	36 (33.0‐39.0)	119 (112.0‐127.0)	66 (62.0‐71.0)
Fruit & vegetable						
Low	(*n* = 376)	35.3 (32.5‐40.0)	103 (96.0‐112.0)	35 (33.0‐39.0)	119 (112.0‐127.0)	66 (62.0‐71.0)
High	(*n* = 77)	34.8 (31.5‐38.9)	103 (95.0‐114.0)	35 (33.0‐40.0)	119 (112.9‐130.0)	65 (61.0‐70.0)
African/Caribbean						
Low	(*n* = 412)	35.1 (32.4‐40.0)	103 (95.5‐112.2)	35 (32.5‐39.0)	118 (112.0‐127.0)	66 (61.5‐71.0)
High	(*n* = 41)	35.3 (32.9‐38.4)	103 (97.1‐112.0)	37 (35.5‐39.3)	120 (114.0‐127.6)	67 (63.4‐72.0)
Processed						
Low	(*n* = 402)	34.8 (32.4‐39.1)	102 (95.3‐111.0)	35 (33.0‐38.9)	118 (112.0‐126.6)	66 (62.0‐71.0)
High	(*n* = 51)	37.0 (34.4‐43.3)	112 (98.8‐121.0)	37 (32.0‐42.0)	122 (114.0‐134.0)	66 (62.0‐72.0)
Snacking						
Low	(*n* = 385)	35.3 (32.4‐39.9)	103 (96.0‐112.0)	36 (33.0‐39.0)	118 (112.0‐127.0)	66 (62.0‐71.0)
High	(*n* = 68)	34.7 (32.0‐39.4)	102 (95.5‐113.5)	35 (32.7‐38.5)	120 (113.0‐126.0)	66 (62.0‐72.0)

*Note*: Women were excluded if currently pregnant or had given birth in the previous 4 months (*n* = 46). Data are presented as median (IQR). Low and high trajectories for the four individual dietary patterns.

Abbreviations: MUA, mid‐upper arm; UPBEAT, UK Pregnancy Better Eating and Activity Trial.

**TABLE 3:** Adjusted association between the dietary trajectory and adiposity outcomes at 3 years post partum in UPBEAT women (*n* = 413) given as adjusted standardized β‐coefficients (95% CI)

	Fruit & vegetable	African/Caribbean	Processed	Snacking
BMI	−0.05 (−0.31 to 0.20)	0.00 (−0.39 to 0.39)	0.38 (0.06 to 0.69)	−0.06 (−0.33 to 0.22)
Waist circumference	0.05 (−0.21 to 0.31)	0.00 (−0.40 to 0.39)	0.35 (0.03 to 0.67)	−0.03 (−0.31 to 0.24)
MUA circumference	0.06 (−0.19 to 0.32)	0.08 (−0.31 to 0.47)	0.36 (0.04 to 0.67)	0.04 (−0.23 to 0.32)
Hip circumference	0.10 (−0.15 to 0.36)	−0.03 (−0.42 to 0.36)	0.30 (−0.01 to 0.62)	0.01 (−0.26 to 0.28)
Thigh circumference	−0.12 (−0.37 to 0.12)	−0.21 (−0.59 to 0.17)	0.20 (−0.10 to 0.52)	0.13 (−0.13 to 0.40)

*Note*: Adjusted for Index of Multiple Deprivation score, parity, ethnicity, physical activity at 3 years post partum, maternal age, and intervention arm. All adiposity outcomes were log transformed before analyses. For ease of interpretation, these values were standardized (mean ± SD: 0 ± 1) to allow comparison between measures. All regression coefficients represent the relative change in the SD of the outcome with the low trajectory for the dietary patterns assigned as the reference group.

Abbreviations: MUA, mid‐upper arm; UBEAT, UK Pregnancy Better Eating and Activity Trial.

## DISCUSSION

To our knowledge, this is the first study to apply latent class modeling to define dietary trajectories across the antenatal and postdelivery periods and describe associations between these trajectories and sociodemographic characteristics and adiposity measures in a cohort of women with obesity. We have shown that dietary patterns defined as diets high in fruit and vegetable, African/Caribbean, processed, and snacking foods track from early pregnancy to 3 years post delivery. We reported associations between several sociodemographic characteristics and these dietary pattern trajectories, including time spent in higher education, smoking, parity, maternal age, ethnicity, and level of neighborhood deprivation. We also found that a processed dietary pattern across pregnancy and 3 years post delivery is associated with higher adiposity, including BMI and circumferences.

We examined the changes in dietary patterns from early pregnancy to 3 years post delivery and found that dietary habits tended toward returning to their earlier dietary pattern score. Interestingly, we found that the average scores for the processed dietary pattern fell slightly during pregnancy, perhaps because of this group of women following a healthier diet while pregnant. The same applied to the women who initially had high snacking diet pattern scores, as the average scores for this dietary pattern also fell during gestation. However, we found no evidence of improved diet among women who consistently had a diet high in fruit and vegetables. These findings may suggest that women with poorer diets become more aware of their food habits while pregnant and subsequently attempt to improve their diet, a change less important for those already habitually eating a healthy diet. A study of a cohort of women with heterogenous BMI found that intakes of caffeine, alcohol, and meats tended to fall during pregnancy and those of fruit, milk, and sweet foods tended to increase [27]. These findings contrast with the data in this study. One explanation may be heightened food insecurity and a potential avoidance of these food types in women with obesity [28].

A common profile in the unhealthy postnatal trajectories (i.e., processed and snacking patterns) was a trend toward their early‐pregnancy dietary pattern. In support of these trajectories, other studies have also shown that diet quality decreases postnatally [29], which is thought to be a result of mothers adapting to the needs of their baby rather than prioritizing their own health. A similar rebound was observed among women who had high adherence to the healthier patterns (fruit & vegetable and African/Caribbean). The sociodemographic characteristics associated with these dietary patterns (e.g., less time spent in education, high neighborhood deprivation) may be a key underlying driver, which also was observed in another postnatal cohort [30], and may be compounded by sociocultural drivers of diet such as social support or family food preferences [31]. These findings highlight the importance of identifying population groups that would benefit from public health interventions and strategies that aim to improve maternal diet postnatally.

The findings of this study show that lower maternal age, less time spent in education, high neighborhood deprivation, and maternal smoking were associated with a dietary pattern of processed foods. Similar findings have been reported in other maternal cohorts [14, 32]. In these studies, the most common characteristics associated with poorer dietary habits were SES, maternal smoking, lower maternal age, and less time spent in education. These factors have been shown to cluster in individuals, and SES is thought to be a critical factor [33]. The relationship between SES and diet quality has been shown to be exacerbated further by disparities between the cost of energy‐dense and nutrient‐dense foods [34] and exposure to unhealthy food environments. Evidence from the UK showed that associations between exposure to fast food outlets or less healthy supermarket environments and unhealthy dietary habits were more pronounced among individuals with lower educational attainment [35]. These findings indicate that individuals of lower SES are disproportionately at risk of poorer diet quality likely because of the limited economic and psychosocial resources available to them [36]. They may also reflect cultural differences in consumption of highly processed foods. Viewed in conjunction with our findings, there is strong evidence for providing support to pregnant and postpartum women from socially deprived backgrounds and for providing effective interventions to improve diet quality and unhealthy food environments.

We have also shown that continued maternal consumption of a diet high in processed foods is associated with a more adipose body composition 3 years after giving birth. The relationship between consumption of energy‐dense/ultraprocessed foods and higher rates of obesity has been well established in both pediatric and adult populations [37, 38]. However, very few studies have assessed longitudinal dietary intake in relationship to adiposity outcomes, and those that have are limited to a single follow‐up visit [39] or to pediatric populations [40]. Our study builds on these findings as we have shown that a diet persistently high in processed foods assessed at five time points across pregnancy and up to 3 years post delivery is associated with higher maternal BMI and adiposity measures compared with those women with a consistently low processed dietary pattern score. Our findings support initiatives that promote the reduction of energy‐dense/ultraprocessed foods from the earliest stages of pregnancy. Previous research in women from disadvantaged areas has shown that interventions that aim to promote behavior change by encouraging 1:1 healthy conversations [41] with trained practitioners can improve self‐efficacy and encourage people to make changes to manage their own health [42]. It is therefore important that public health initiatives that aim to improve overall diet quality across the antenatal and postnatal period deliver behavior change interventions focused on the individual. The postnatal period is also the preconception period for any subsequent pregnancies, so any improvements in nutrition and lifestyle preconceptionally could influence maternal weight trajectories across the reproductive years.

The data presented in this paper are from a prospective cohort of women with obesity, recruited from ethnically diverse and predominantly socially deprived backgrounds. The in‐depth data collection throughout pregnancy and postnatally provides a comprehensive data set, including maternal demographic and social characteristics that allowed for a detailed analysis of the association between longitudinal dietary intake and adiposity outcomes. Limitations include attrition of the study population from the original cohort, although the sample size was large enough to provide an appropriate comparison at 3 years post delivery; attrition can introduce either selection or collider bias to the results [43]. Another consideration is the observational study design, in which causal inferences are more difficult; in order to minimize bias, we used a direct acyclic graph to identify the confounders that should be included in the models; furthermore, the assessment of body composition was limited to BMI and circumferences, which were not validated against gold standard techniques. The dietary patterns obtained using factor analysis involved several arbitrary decisions, including the final number of factors and rotation methods [44], and FFQ data have previously been associated with recall bias [45]. Reverse causation is also a consideration because maternal adiposity may also influence the woman's diet. Finally, GBTM also has limitations compared with other latent class modeling techniques [15]. GBTM assumes that individuals within a class are homogenous [21], whereas GMM allows for varying interindividual differences within a class. However, as discussed in *Results*, we found a strong agreement between class assignment in the two methods.

## CONCLUSION

Maternal prepregnancy obesity and excessive weight gain during the pregnancy and post‐delivery periods can increase rates of obesity in women and alter their weight gain trajectory across the life course. We have shown that dietary habits track from early pregnancy to 3 years post delivery, and women who consistently adhered to a diet high in processed foods (including crisps, chips, processed meat products, and fizzy drinks) had higher measures of adiposity, including BMI and arm and waist circumferences 3 years after giving birth compared with those who did not follow a processed dietary trajectory. We also identified several sociodemographic characteristics associated with the high intake of processed foods, including being of a younger age, being of White ethnicity, living in more disadvantaged communities, and smoking in pregnancy. This study supports the development of policies that aim to improve unhealthy food environments, particularly in more deprived or socioeconomically disadvantaged areas, as well as public health strategies focused on improving nutrition behaviors before and between pregnancies [46]. These initiatives are likely to support women with obesity to improve dietary habits during these critical time points and help reduce the risk of gaining excess weight during the reproductive years.

## AUTHOR CONTRIBUTIONS

Study design: Angela C. Flynn, Paul T. Seed, and Lucilla Poston; data collection: Angela C. Flynn and Paul T. Seed; data analysis: Kathryn V. Dalrymple; data interpretation: Kathryn V. Dalrymple, Christina Vogel, Angela C. Flynn, Keith M. Godfrey, Lucilla Poston, Hazel M. Inskip, and Sarah R. Crozier; supervision: Christina Vogel, Hazel M. Inskip, and Sarah R. Crozier; writing—original draft preparation and project administration: Kathryn V. Dalrymple. All authors were involved in writing the paper and had final approval of the submitted and published versions.

## FUNDING INFORMATION

Kathryn V. Dalrymple is funded by the Medical Research Council (MRC) (grant number: MR/V005839/1). Keith M. Godfrey is supported by the National Institute for Health Research (NIHR Senior Investigator [NF‐SI‐0515‐10042] and the NIHR Southampton Biomedical Research Centre), the European Union (Erasmus+ Programme ImpENSA 598488‐EPP‐1‐2018‐1‐DE‐EPPKA2‐CBHE‐JP), and the British Heart Foundation (RG/15/17/3174). UPBEAT was supported by the European Union's Seventh Framework Programme (FP7/2007–2013), project Early Nutrition, grant agreement no. 289346, and the NIHR (UK) Programme Grants for Applied Research Programme (RP‐0407‐10452). Support was also provided by the Chief Scientist Office Scotland, Guy's and St Thomas' Charity, and Tommy's Charity (registered charity no. 1060508). Lucilla Poston is funded by Tommy's Charity. Paul T. Seed is partly funded by King's Health Partners Institute of Women and Children's Health (KHP) and ARC South London (NIHR). Lucilla Poston is an NIHR Senior Investigator Emeritus (NI‐SI‐0512‐10104). For the purpose of open access, the authors have applied a Creative Commons Attribution (CC BY) license to any author accepted manuscript version arising from this submission.

## CLINICAL TRIAL REGISTRATION

ISCRTN89971375.

## CONFLICT OF INTEREST

Keith M. Godfrey has received reimbursement for speaking at conferences sponsored by companies selling nutritional products and is part of an academic consortium that has received research funding from Abbott Nutrition, Nestec, BenevolentAI Bio Ltd., and Danone. The other authors declared no conflict of interest.

## Supporting information


**Data S1.** Supporting Information.

## Data Availability

The data presented in this study are available on request from the corresponding author following approval from the UPBEAT consortium.

## References

[1] Hruby A , Hu FB . The epidemiology of obesity: a big picture. Pharmacoeconomics. 2015;33:673‐689. 25471927 10.1007/s40273-014-0243-x PMC4859313

[2] Catalano PM , Shankar K . Obesity and pregnancy: mechanisms of short term and long term adverse consequences for mother and child. Br Med J. 2017;356:j1. doi:10.1136/bmj.j1 28179267 PMC6888512

[3] Relph S , NMPA Project Team . NHS maternity care for women with a body mass index of 30 kg/m^2^ or above: births between 1 April 2015 and 31 March 2017 in England, Wales and Scotland. Royal College of Obstetricians and Gynaecologists; 2021.

[4] Dalrymple KV , El‐Heis S , Godfrey KM . Maternal weight and gestational diabetes impacts on child health. Curr Opin Clin Nutr Metab Care. 2022;25:203‐208. 35199660 10.1097/MCO.0000000000000826 PMC7612944

[5] Poston L , Caleyachetty R , Cnattingius S , et al. Preconceptional and maternal obesity: epidemiology and health consequences. Lancet Diabetes Endocrinol. 2016;4:1025‐1036. 27743975 10.1016/S2213-8587(16)30217-0

[6] Dalrymple KV , Thompson JMD , Begum S , et al. Relationships of maternal body mass index and plasma biomarkers with childhood body mass index and adiposity at 6 years: the children of SCOPE study. Pediatr Obes. 2019;14:e12537. doi:10.1111/ijpo.12537 31232532 PMC6731120

[7] Public Health England . Health matters: obesity and the food environment. Published March 31, 2017. https://www.gov.uk/government/publications/health‐matters‐obesity‐and‐the‐food‐environment/health‐matters‐obesity‐and‐the‐food‐environment‐‐2

[8] Lakerveld J , Mackenbach J . The upstream determinants of adult obesity. Obes Facts. 2017;10:216‐222. 28564658 10.1159/000471489 PMC5644962

[9] Smith M , Hosking J , Woodward A , et al. Systematic literature review of built environment effects on physical activity and active transport ‐ an update and new findings on health equity. Int J Behav Nutr Phys Act. 2017;14:158. doi:10.1186/s12966-017-0613-9 29145884 PMC5693449

[10] Rauber F , Steele EM , Louzada ML d C , Millett C , Monteiro CA , Levy RB . Ultra‐processed food consumption and indicators of obesity in the United Kingdom population (2008–2016). PLoS One. 2020;15:e0232676. doi:10.1371/journal.pone.0232676 32357191 PMC7194406

[11] Ashman AM , Collins CE , Hure AJ , Jensen M , Oldmeadow C . Maternal diet during early childhood, but not pregnancy, predicts diet quality and fruit and vegetable acceptance in offspring: maternal diet and diet quality in offspring. Matern Child Nutr. 2016;12:579‐590. 25294406 10.1111/mcn.12151 PMC6860109

[12] McKinley M , Allen‐Walker V , McGirr C , Rooney C , Woodside J . Weight loss after pregnancy: challenges and opportunities. Nutr Res Rev. 2018;31:225‐238. 10.1017/S0954422418000070 29984681

[13] Sotres‐Alvarez D , Herring AH , Siega‐Riz A‐M . Latent transition models to study Women's changing of dietary patterns from pregnancy to 1 year postpartum. Am J Epidemiol. 2013;177:852‐861. 23538942 10.1093/aje/kws303 PMC3668424

[14] Lee YQ , Colega M , Sugianto R , et al. Tracking of dietary patterns between pregnancy and 6 years post‐pregnancy in a multiethnic Asian cohort: the growing up in Singapore towards healthy outcomes (GUSTO) study. Eur J Nutr. 2021;61:985‐1001. 34686887 10.1007/s00394-021-02703-z PMC7612407

[15] van der Nest G , Lima Passos V , Candel MJJM , van Breukelen GJP . An overview of mixture modelling for latent evolutions in longitudinal data: modelling approaches, fit statistics and software. Adv Life Course Res. 2020;43:100323. doi:10.1016/j.alcr.2019.100323 36726256

[16] Poston L , Bell R , Croker H , et al. Effect of a behavioural intervention in obese pregnant women (the UPBEAT study): a multicentre, randomised controlled trial. Lancet Diabetes Endocrinol. 2015;3:767‐777. 26165396 10.1016/S2213-8587(15)00227-2

[17] Payne RA , Abel GA . UK indices of multiple deprivation‐a way to make comparisons across constituent countries easier. Health Stat Q. 2012;53. https://webarchive.nationalarchives.gov.uk/ukgwa/20160107054137/ http://www.ons.gov.uk/ons/rel/hsq/health‐statistics‐quarterly/no–53–spring‐2012/uk‐indices‐of‐multiple‐deprivation.html

[18] Bingham SA , Gill C , Welch A , et al. Validation of dietary assessment methods in the UK arm of EPIC using weighed records, and 24‐hour urinary nitrogen and potassium and serum vitamin C and carotenoids as biomarkers. Int J Epidemiol. 1997;26(suppl 1):S137‐S151. 9126542 10.1093/ije/26.suppl_1.s137

[19] Flynn AC , Seed PT , Patel N , et al. Dietary patterns in obese pregnant women; influence of a behavioral intervention of diet and physical activity in the UPBEAT randomized controlled trial. Int J Behav Nutr Phys Act. 2016;13:124. doi:10.1186/s12966-016-0450-2 27894316 PMC5126873

[20] Northstone K , Emmett PM . A comparison of methods to assess changes in dietary patterns from pregnancy to 4 years post‐partum obtained using principal components analysis. Br J Nutr. 2008;99:1099‐1106. 17916275 10.1017/S0007114507842802 PMC2493053

[21] Nagin DS . Group‐based trajectory modeling: an overview. Ann Nutr Metab. 2014;65:205‐210. 25413659 10.1159/000360229

[22] Nagin D . Group‐Base Modeling of Development. Harvard University Press; 2005.

[23] Louvet B , Gaudreau P , Menaut A , Genty J , Deneuve P . Longitudinal patterns of stability and change in coping across three competitions: a latent class growth analysis. J Sport Exerc Psychol. 2007;29:100‐117. 17556778 10.1123/jsep.29.1.100

[24] van de Schoot R , Sijbrandij M , Winter SD , Depaoli S , Vermunt JK . The GRoLTS‐checklist: guidelines for reporting on latent trajectory studies. Struct Equ Modeling. 2017;24:451‐467.

[25] Muthén B . Second‐generation structural equation modeling with a combination of categorical and continuous latent variables: new opportunities for latent class–latent growth modeling. In: Collins LM, Sayer AG, eds. New Methods for the Analysis of Change. American Psychological Association; 2001:291‐322.

[26] Dalrymple KV , Tydeman FAS , Taylor PD , et al. Adiposity and cardiovascular outcomes in three‐year‐old children of participants in UPBEAT, an RCT of a complex intervention in pregnant women with obesity. Pediatr Obes. 2021;16(3):e12725. doi:10.1111/ijpo.12725 32914569 PMC7116719

[27] Forbes LE , Graham JE , Berglund C , Bell RC . Dietary change during pregnancy and women's reasons for change. Nutrients. 2018;10:1032. doi:10.3390/nu10081032 30096771 PMC6115730

[28] Laraia B , Vinikoor‐Imler LC , Siega‐Riz AM . Food insecurity during pregnancy leads to stress, disordered eating, and greater postpartum weight among overweight women. Obesity (Silver Spring). 2015;23:1303‐1311. 25959858 10.1002/oby.21075 PMC6563905

[29] Lee YQ , Loh J , Ang RSE , Chong MF‐F . Tracking of maternal diet from pregnancy to postpregnancy: a systematic review of observational studies. Curr Dev Nutr. 2020;4:nzaa118. doi:10.1093/cdn/nzaa118 32793849 PMC7408223

[30] Tovar A , Kaar JL , McCurdy K , Field AE , Dabelea D , Vadiveloo M . Maternal vegetable intake during and after pregnancy. BMC Pregnancy Childbirth. 2019;19:267. doi:10.1186/s12884-019-2353-0 31349808 PMC6660649

[31] Ruddock HK , Brunstrom JM , Vartanian LR , Higgs S . A systematic review and meta‐analysis of the social facilitation of eating. Am J Clin Nutr. 2019;110:842‐861. 31435639 10.1093/ajcn/nqz155

[32] de Castro MBT , Freitas Vilela AA , de Oliveira ASD , et al. Sociodemographic characteristics determine dietary pattern adherence during pregnancy. Public Health Nutr. 2016;19:1245‐1251. 26400675 10.1017/S1368980015002700 PMC10270904

[33] Darmon N , Drewnowski A . Does social class predict diet quality? Am J Clin Nutr. 2008;87:1107‐1117. 18469226 10.1093/ajcn/87.5.1107

[34] Drewnowski A , Darmon N . The economics of obesity: dietary energy density and energy cost. Am J Clin Nutr. 2005;82(suppl 1):265S‐273S. 16002835 10.1093/ajcn/82.1.265S

[35] Vogel C , Lewis D , Ntani G , et al. The relationship between dietary quality and the local food environment differs according to level of educational attainment: a cross‐sectional study. PLoS One. 2017;12:e0183700. doi:10.1371/journal.pone.0183700 28841678 PMC5571951

[36] Adams J , Mytton O , White M , Monsivais P . Why are some population interventions for diet and obesity more equitable and effective than others? The role of individual agency. PLoS Med. 2016;13:e1001990. doi:10.1371/journal.pmed.1001990 27046234 PMC4821622

[37] Johnson L , Mander A , Jones L , Emmett P , Jebb S . Energy‐dense, low‐fiber, high‐fat dietary pattern is associated with increased fatness in childhood. Am J Clin Nutr. 2008;87:846‐854. 18400706 10.1093/ajcn/87.4.846

[38] Askari M , Heshmati J , Shahinfar H , Tripathi N , Daneshzad E . Ultra‐processed food and the risk of overweight and obesity: a systematic review and meta‐analysis of observational studies. Int J Obes (Lond). 2020;44:2080‐2091. 32796919 10.1038/s41366-020-00650-z

[39] Canhada SL , Luft VC , Giatti L , et al. Ultra‐processed foods, incident overweight and obesity, and longitudinal changes in weight and waist circumference: the Brazilian Longitudinal Study of Adult Health (ELSA‐Brasil). Public Health Nutr. 2020;23:1076‐1086. 31619309 10.1017/S1368980019002854 PMC7282862

[40] Dalrymple KV , Vogel C , Godfrey KM , et al. Longitudinal dietary trajectories from preconception to mid‐childhood in women and children in the Southampton Women's Survey and their relation to offspring adiposity: a group‐based trajectory modelling approach. Int J Obes (Lond) 2022;46:758‐766. 34916617 10.1038/s41366-021-01047-2 PMC8960403

[41] Lawrence W , Black C , Tinati T , et al. ‘Making every contact count’: evaluation of the impact of an intervention to train health and social care practitioners in skills to support health behaviour change. J Health Psychol. 2016;21:138‐151. 24713156 10.1177/1359105314523304 PMC4678584

[42] Lawrence W , Watson D , Barker H , Vogel C , Rahman E , Barker M . Meeting the UK Government's prevention agenda: primary care practitioners can be trained in skills to prevent disease and support self‐management. Perspect Public Health. 2021;142:158‐166. 33588652 10.1177/1757913920977030 PMC9047100

[43] Rohrer JM . Thinking clearly about correlations and causation: graphical causal models for observational data. Adv Methods Pract Psychol Sci. 2018;1:27‐42.

[44] Costello AB , Osborne JW . Best practices in exploratory factor analysis: four recommendations for getting the most from your analysis. Practical Assessment, Research, and Evaluation. 2005;10:7. doi:10.7275/jyj1-4868

[45] Martínez ME , Marshall JR , Sechrest L . Invited commentary: factor analysis and the search for objectivity. Am J Epidemiol. 1998;148:17‐19. 9663398 10.1093/oxfordjournals.aje.a009552

[46] Stephenson J , Heslehurst N , Hall J , et al. Before the beginning: nutrition and lifestyle in the preconception period and its importance for future health. Lancet. 2018;391:1830‐1841. 29673873 10.1016/S0140-6736(18)30311-8 PMC6075697

