# Supplementary material for: Longitudinal dietary trajectories from pregnancy to 3 years post delivery in women with obesity: relationships with adiposity
Source: Obesity (Silver Spring). 2023 Mar 6;31(4):1159–69. doi: 10.1002/oby.23706 (PMC10947498; doi:10.1002/oby.23706)
Supplement: Supplementary file 1 — Data S1. Supporting Information. [file OBY-31-1159-s001.docx]

**Supplementary Tables and Figures**

Figure S1: DAG for dietary pattern (exposure) and maternal adiposity measures at 3-years post-delivery


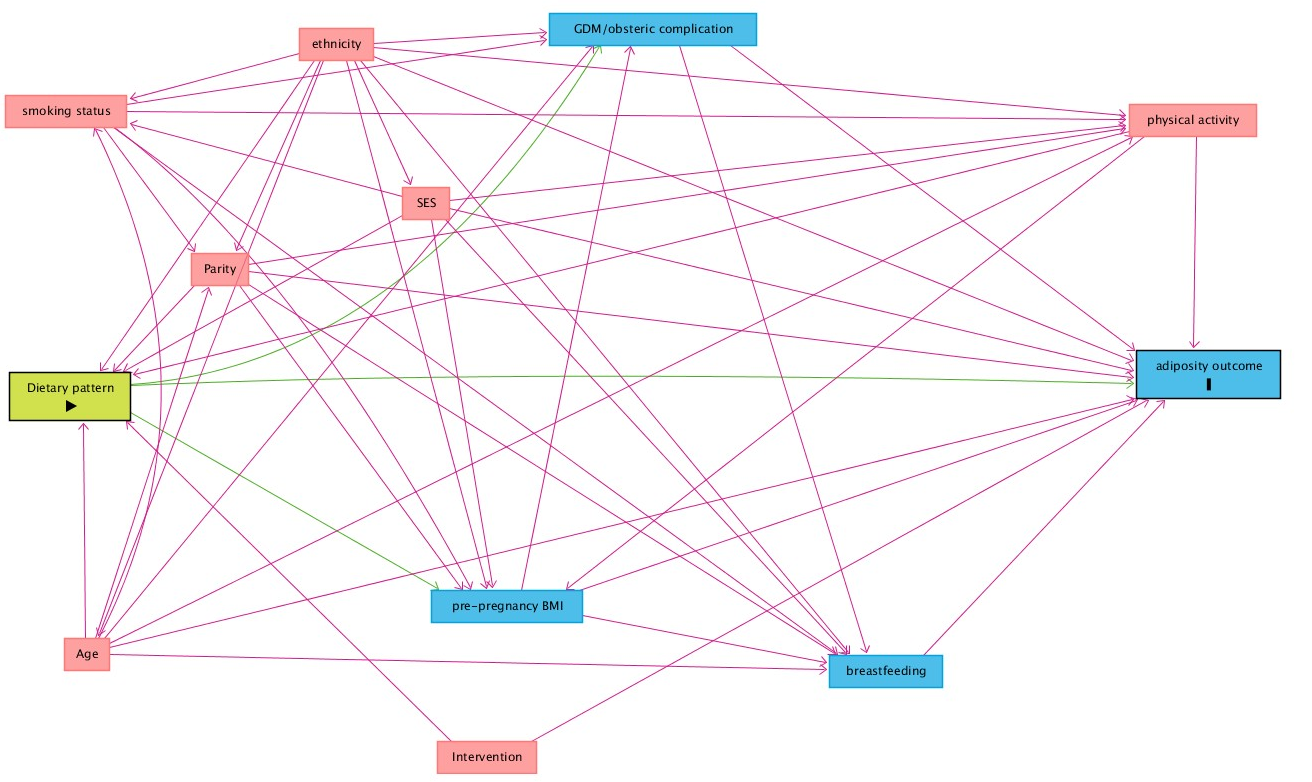


*confounders adjusted for in the analysis are index of multiple deprivation score, parity, ethnicity, physical activity maternal age and intervention arm*

| Table S1: Guidelines for Reporting on Latent Trajectory Studies (GRoLT) checklist | |
| --- | --- |
| Item 1: metric of time | Age in weeks from baseline (0, 10, 20, 52 and 182 weeks) |
| Item 2: fixed or varying occasion | 5 fixed time points, baseline (15-18 weeks gestation), 26-28 weeks gestation, 34-36 weeks gestation, 6 months and 3 years post-delivery. |
| Item 3a: missing data mechanism | Missing at random, there was available data at the following timepoints: baseline (15-18 weeks gestation) n=1018, 26-28 weeks gestation n=853, 34-36 weeks gestation n=686, 6 months post-delivery n=431, 3 years post-delivery n=504. |
| Item 3b: auxiliary variables | Supplementary Table 2 reports demographic data of those lost to follow-up. Participants who completed the 3 year follow-up were more likely to have a greater number of years in education, less likely to smoke, higher maternal age and lower BMI at baseline, more likely to be nulliparous and more likely to be of white ethnicity. |
| Item 3c: how dealt with missing data | If the mother was missing all data points (n=346) these participants were excluded from the analysis. |
| Item 4: distribution | Censored normal distribution |
| Item 5: software | Stata 15.0 'traj' command |
| Item 6a: LGMM versus LCGA | Our findings for the LCGA were compared with the output of a LGMM. |
| Item 6b: across-class variance-covariance matrix | Default |
| Item 7: functional form | Intercept, linear, quadratic and cubic |
| Item 8: covariates | Covariates were not included in the model to predict trajectory groups |
| Item 9: random starts | Default |
| Item 10: model comparison | Average Posterior Probability Assignment, Bayesian Information Criterion, odds of correct classification, entropy, percentage of participants assigned to each group |
| Item 11: 1-class solution | Supplementary table 3-6 |
| Item 12: sample size per class | Supplementary table 3-6 |
| Item 13: entropy | 0.7-0.92 |
| Item 14a: plot of final solution | Figure 2 |
| Item 14b: plots for each model | Supplementary figure 2 |
| Item 14c: plots of individual trajectories | Supplementary figure 3-6 |
| Item 15: descriptive statistics | Table 1 |
| Item 16: syntax | traj, var(fruitandveg_*) indep(t_*) model(cnorm) order(0 3) min(-4) max(5) |

| **Table S2: Dietary trajectories, stratified by randomisation arm.** | | |
| --- | --- | --- |
| **traj group membership stratified by randomisation arm for the Fruit and Veg pattern** | | |
| Intervention (n=604) | Group membership | P* |
| 2 classes | (1) 83.4%, n=504  (2) 16.6% n= 100 |  |
| Control (n=604) | |  |
| 2 classes | (1) 83.4%, n=504  (2) 16.6%, n=100 | 1.00 |
| traj group membership stratified by randomisation arm for the Cultural pattern | | |
| Intervention (n=604) | Group membership |  |
| 2 classes | (1) 90.0%, n=544  (2) 10.0% n= 60 |  |
| Control (n=604) |  |  |
| 2 classes | (1) 86.4%, n=522  (2) 13.6%, n=82 | 0.05 |
| traj group membership stratified by randomisation arm for the Processed pattern | | |
| Intervention (n=604) | Group membership |  |
| 2 classes | (1) 85.6%, n=517  (2) 14.4% n= 87 |  |
| Control (n=604) |  |  |
| 2 classes | (1) 88.4%, n=528  (2) 12.6%, n=76 | 0.354 |
| traj group membership stratified by randomisation arm for the Snacking pattern | | |
| Intervention (n=604) | Group membership |  |
| 2 classes | (1) 85.6%, n=517  (2) 14.4% n= 87 |  |
| Control (n=604) |  |  |
| 2 classes | (1) 83.4%, n=504  (2) 16.6%, n=100 | 0.301 |

| **Table S3: Demographic characteristics of all UPBEAT participants vs those included in the final analysis (n=413)** | | | | | | | |
| --- | --- | --- | --- | --- | --- | --- | --- |
| **Maternal** | | | **Attended the 3-year follow-up n=413** | | **Didn’t attend the 3-year follow-up n=1141** | **Total UPBEAT cohort n=1554** | **P-values for difference between those who were followed-up (n=413) vs those who were not (n=1141)** *^b^* |
|  | | | |  | **Mean (SD)/ Median (IQR) / N (%) ^a^** | |  |
| Body mass index (kg/m^2^) | | | | 34.7 (32.5-37.7) | 35.4 (32.9-38.8) | 35.1 (32.8-28.5) | 0.005 |
| Years in full time education | | | | 15.2 (2.7) | 14.6 (2.9) | 14.8 (2.8) | <0.001 |
| Ethnicity | White  Black  Asian  Other | | | 279 (68%)  95 (23%)  18 (4%)  21 (5%) | 694 (61%)  306 (27%)  77 (7%)  64 (6%) | 973 (63%)  401 (26%)  95 (5%) 85 (6%) | 0.07 |
| Smoking in pregnancy | | | | 53 (13%) | 200 (18%) | 253 (16%) | 0.03 |
| Parity (Nulliparous) | | | | 202 (49%) | 472 (41%) | 674 (43%) | 0.008 |
| Age at baseline (years) | | | | 31.3 (5.2) | 30.2 (5.6) | 30.5 (5.5) | <0.001 |
| IMD | | Least deprived  2  3  4  Most deprived | | 25 (6%)  32 (8%)  51 (12%)  144 (35%)  161 (39%) | 40 (4%)  71 (6%) 126 (11%) 389 (34%)  509 (45%) | 65 (4%)  103 (7%)  177 (11%)  533 (34%)  670 (43%) | 0.07 |
| Baseline dietary pattern score | | Fruit and vegetable | | -0.24 (-0.52, 0.29) | -0.19 (-0.55, 0.37) | -0.20 (-0.54, 0.34) | 0.73 |
|  |  | Cultural | | -0.25 (-0.44, -0.02) | -0.22 (-0.42, 0.12) | -0.23 (-0.43, 0.08) | 0.18 |
|  |  | Processed | | -0.11 (-0.40, 0.25) | -0.07 (-0.38, 0.24) | -0.08 (-0.39, 0.25) | 0.53 |
|  |  | Snacking | | -0.11 (-0.46, 0.30) | -0.16 (-0.54, 0.32) | -0.18 (-0.51, 0.32) | 0.18 |
| *Abbreviations: IQR: Interquartile range, N: number; SD: standard deviation.* **^a^** *Binary and categorical variables are presented using counts and percentages. The distribution of continuous variables was assessed using coefficients of skewness and then summarized by mean and standard deviation or median and interquartile range where appropriate.  ^b^ Differences between those who attended the 3-year follow-up vs those who did not was conducted by t-test or Mann–Whitney rank-sum test for continuous variables and χ2-test for categorical variables.* | | | | | | | |

| **Table S4: A description of the dietary patterns at each timepoint, stratified by randomisation arm** | | | | | | | | |
| --- | --- | --- | --- | --- | --- | --- | --- | --- |
|  | **Intervention [median (IQR)]** | | | | **Control[median (IQR)]** | | | |
|  | F&V | Cultural | Processed | Snacking | F&V | Cultural | Processed | Snacking |
| Baseline  (n=512, 496) | -0.21 (-0.55, 0.30) | -0.21 (-0.42, 0.11) | -0.07 (-0.38, 0.26) | -0.15 (-0.51, 0.34) | -0.19 (-0.54, 0.38) | -0.24 (-0.43, 0.06) | -0.12 (-0.40, 0.22) | -0.14 (-0.49, 0.29) |
| 28 weeks’  (n=411, 433) | -0.23 (-0.56, 0.26) | -0.22 (-0.42, 0.07) | -0.21 (-0.43, 0.06) | -0.29 (-0.58, 0.14) | -0.25 (-0.61, 0.20) | -0.24 (-0.42, 0.02) | -0.11 (-0.38, 0.24) | -0.06 (-0.44, 0.38) |
| 36 weeks’  (n=318, 359) | -0.25 (-0.60, 0.16) | -0.24 (-0.43, 0.02) | -0.20 (-0.44, 0.07) | -0.28 (-0.55, 0.11) | -0.29 (-0.65, 0.12) | -0.26 (-0.43, -0.02) | -0.13 (-0.38, 0.16) | -0.14 (-0.49, 0.34) |
| 6 months  (n=205, 217) | -0.26 (-0.60, 0.17) | -0.14 (-0.37, 0.10) | -0.12 (-0.42, 0.18) | -0.15 (-0.48, 0.26) | -0.26 (-0.56, 0.11) | -0.22 (-0.36, 0.01) | -0.16 (-0.40, 0.20) | -0.05 (0.39, 0.36) |
| 3 years  (n=240, 252) | -0.19 (-0.48, 0.46) | -0.18 (-0.36, 0.06) | -0.16 (-0.40, 0.15) | -0.26 (-0.56, 0.12) | -0.21 (-0.58, 0.42) | -0.18 (0.33, 0.12) | -0.13 (-0.37, 0.19) | -0.03 (-0.43,0.41) |

| **Table S5: traj criteria statistics for 1 to 3 trajectories for fruit and vegetable pattern; (n=1208)** | | | | | | |
| --- | --- | --- | --- | --- | --- | --- |
|  | **BIC** | **Group membership** | **APPA** | **Entropy** | **OCC** |  |
| 1 class  (3) | -3925 | (1) 100% | - | - | - |  |
| **2 classes**  **(0 3)** | **-3535** | **(1) 83%**  **(2) 17%** | **96%**  **89%** | **0.81** | **5.3**  **36.2** |  |
| 3 classes  (0 3 0) | -3441 | (1) 75%  (2) 22%  (3) 3% | 93%  83%  93% | 0.80 | 4.7  15.7  423.3 |  |
| *Abbreviations: APPA: Average Posterior Probability Assignment, BIC: Bayesian Information Criteria, OCC: odds of correct classification.* | | | | | | |

| **Table S6: traj criteria statistics for 1 to 3 trajectories for African/Caribbean; (n=1208)** | | | | | | |
| --- | --- | --- | --- | --- | --- | --- |
|  | **BIC** | **Group membership** | **APPA** | **Entropy** | **OCC** |  |
| 1 class  (3) | -2983 | (1) 100% | - | - | - |  |
| **2 classes**  **(1 3)** | **-2176** | **(1) 88%**  **(2) 12%** | **99%**  **94%** | **0.93** | **10.0**  **122.4** |  |
| 3 classes  (0 1 3) | -1948 | (1) 79%  (2) 16%  (3) 5% | 97%  85%  95% | 0.88 | 8.1  30.1  303.3 |  |
| *Abbreviations: APPA: Average Posterior Probability Assignment, BIC: Bayesian Information Criteria, OCC: odds of correct classification.* | | | | | | |

| **Table S7: traj criteria statistics for 1 to 3 trajectories for processed: (n=1208)** | | | | | | |
| --- | --- | --- | --- | --- | --- | --- |
|  | **BIC** | **Group membership** | **APPA** | **Entropy** | **OCC** |  |
| 1 class  (3) | -2846 | (1) 100% | - | - | - |  |
| **2 classes**  **(0 3)** | **-2580** | **(1) 87%**  **(2) 13%** | **95%**  **85%** | **0.78** | **3.4**  **32.1** |  |
| 3 classes  ( 0 0 2) | -2461 | (1) 60%  (2) 36%  (3) 3% | 83%  81%  92% | 0.66 | 3.7  6.4  300.5 |  |
| *Abbreviations: APPA: Average Posterior Probability Assignment, BIC: Bayesian Information Criteria, OCC: odds of correct classification.* | | | | | | |

| **Table S8: traj criteria statistics for 1 to 3 trajectories for snacking: (n=1208)** | | | | | | |
| --- | --- | --- | --- | --- | --- | --- |
|  | **BIC** | **Group membership** | **APPA** | **Entropy** | **OCC** |  |
| 1 class  (3) | -3619 | (1) 100% | - | - | - |  |
| **2 classes**  **(0 3)** | **-3311** | **(1) 84%**  **(2) 16%** | **96%**  **84%** | **0.79** | **4.5**  **27.0** |  |
| 3 classes  (0 0 2) | -3190 | (1) 66%  (2) 30%  (3) 4% | 88%  82%  94% | 0.71 | 4.1  9.2  347.4 |  |
| *Abbreviations: APPA: Average Posterior Probability Assignment, BIC: Bayesian Information Criteria, OCC: odds of correct classification.* | | | | | | |

| **Table S9: GLLAMM criteria statistics for 1 to 3 trajectories for fruit and veg pattern; (n=1208)** | | | | | | |
| --- | --- | --- | --- | --- | --- | --- |
|  | **BIC** | **Group membership** | **APPA** | **Entropy** | **OCC** |  |
| 1 class | 7844 | (1) 100% | - | - | - |  |
| **2 classes** | **7098** | **(1) 82%**  **(2) 18%** | **96%**  **90%** | **0.85** | **35.7**  **76.6** |  |
| 3 classes | 6891 | (1) 75%  (2) 23%  (3) 3% | 95%  87%  94% | 0.84 | 23.1  43.5  836.6 |  |
| *Abbreviations: APPA: Average Posterior Probability Assignment, BIC: Bayesian Information Criteria, OCC: odds of correct classification.* | | | | | | |

| **Table S10: GLLAMM criteria statistics for 1 to 3 trajectories for African/Caribbean; (n=1208)** | | | | | | |
| --- | --- | --- | --- | --- | --- | --- |
|  | **BIC** | **Group membership** | **APPA** | **Entropy** | **OCC** |  |
| 1 class | 5954 | (1) 100% | - | - | - |  |
| **2 classes** | **4383** | **(1) 89%**  **(2) 11%** | **99%**  **95%** | **0.95** | **92.6**  **283.4** |  |
| 3 classes | 3945 | (1) 81%  (2) 14%  (3) 5% | 98%  88%  95% | 0.92 | 52.5  78.5  740.1 |  |
| *Abbreviations: APPA: Average Posterior Probability Assignment, BIC: Bayesian Information Criteria, OCC: odds of correct classification.* | | | | | | |

| **Table S11: GLLAMM criteria statistics for 1 to 3 trajectories for processed: (n=1208)** | | | | | | |
| --- | --- | --- | --- | --- | --- | --- |
|  | **BIC** | **Group membership** | **APPA** | **Entropy** | **OCC** |  |
| 1 class | 5687 | (1) 100% | - | - | - |  |
| **2 classes** | **5187** | **(1) 87%**  **(2) 13%** | **96%**  **87%** | **0.82** | **26.4**  **70.9** |  |
| 3 classes | 4969 | (1) 61%  (2) 37%  (3) 3% | 87%  85%  94% | 0.72 | 12.8  18.8  910.8 |  |
| *Abbreviations: APPA: Average Posterior Probability Assignment, BIC: Bayesian Information Criteria, OCC: odds of correct classification.* | | | | | | |

| **Table S12: GLLAMM criteria statistics for 1 to 3 trajectories for snacking: (n=1208)** | | | | | | |
| --- | --- | --- | --- | --- | --- | --- |
|  | **BIC** | **Group membership** | **APPA** | **Entropy** | **OCC** |  |
| 1 class | 7226 | (1) 100% | - | - | - |  |
| **2 classes** | **6638** | **(1) 85%**  **(2) 15%** | **97%**  **87%** | **0.83** | **29.8**  **63.0** |  |
| 3 classes | 6416 | (1) 66%  (2) 31%  (3) 3% | 91%  85%  95% | 0.77 | 16.7  24.6  1071.2 |  |
| *Abbreviations: APPA: Average Posterior Probability Assignment, BIC: Bayesian Information Criteria, OCC: odds of correct classification.* | | | | | | |

| **Table S13: Cross tabulation of GBTM with GMM, including spearman’s correlation coefficient** | | | | | | | | | |
| --- | --- | --- | --- | --- | --- | --- | --- | --- | --- |
| Traj  GLLAMM | | Fruit and veg (n) | | Cultural (n) | | Processed (n) | | Snacking (n) | |
|  |  | Low | High | Low | High | Low | High | Low | High |
| Fruit and veg  (0.985) | Low | 999 | 3 | - | - | - | - | - | - |
|  | High | 2 | 204 | - | - | - | - | - | - |
| Cultural  (0.996) | Low | - | - | 1064 | 1 | - | - | - | - |
|  | High | - | - | 0 | 141 | - | - | - | - |
| Processed  (0.982) | Low | - | - | - | - | 1051 | 5 | - | - |
|  | High | - | - | - | - | 0 | 152 | - | - |
| Snacking  (0.975) | Low | - | - | - | - | - | - | 1019 | 8 |
|  | High | - | - | - | - | - | - | 0 | 181 |

| **Table S14: cross tabulation of the fruit and veg trajectories (GBTM) with the three other dietary pattern trajectories** | | |
| --- | --- | --- |
|  | Fruit and Veg (low) | Fruit and veg (high) |
| African/Caribbean (low) | 893 (89%) | 172 (83%) |
| African/Caribbean (high) | 108 (11%) | 34 (16%) |
|  |  |  |
| Processed (low) | 874 (87%) | 177 (86%) |
| Processed (high) | 127 (13%) | 30 (14%) |
|  |  |  |
| Snacks (low) | 859 (86%) | 169 (77%) |
| Snacks (high) | 142 (14%) | 47 (23%) |

Figure S2: The class 1 trajectories for each of the dietary patterns
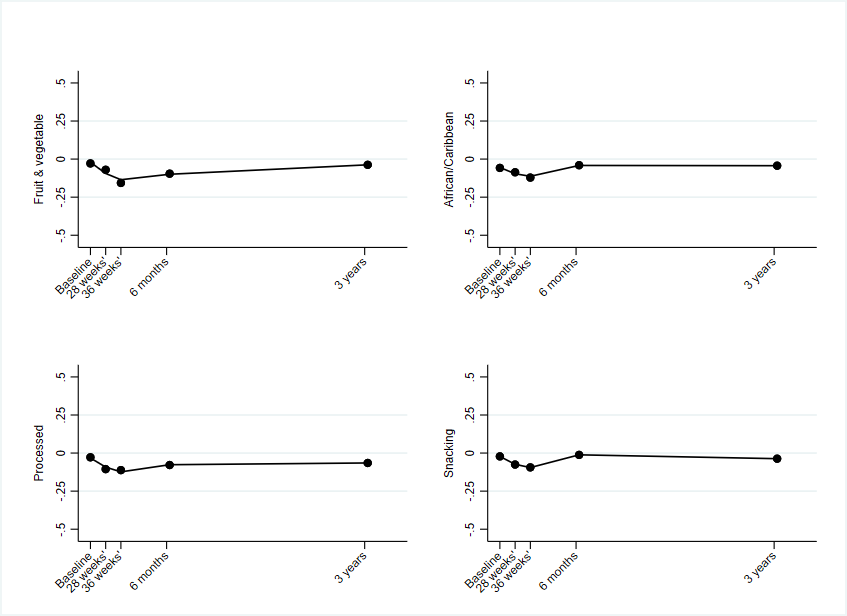


Figure S3: The individual trajectories
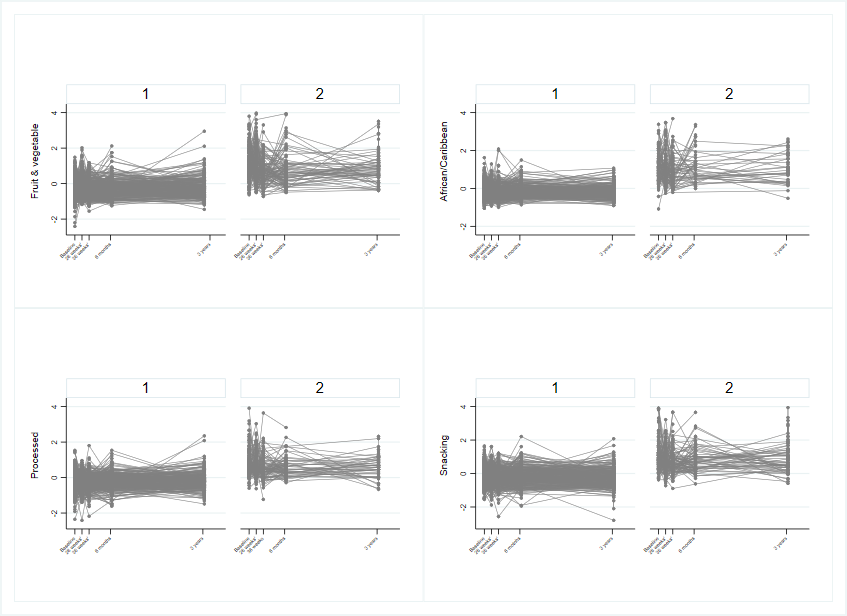
for the two-class models for each of the dietary patterns
